# Supplementary material for: Identification of photoperiod-induced specific miRNAs in the adrenal glands of Sunite sheep (Ovis aries)
Source: Front Vet Sci. 2022 Jul 22;9:888207. doi: 10.3389/fvets.2022.888207 (PMC9354845; doi:10.3389/fvets.2022.888207)
Supplement: Supplementary file 1 [file Data_Sheet_1.ZIP › Supplementary file/miRDeep2 file.zip!/miRDeep2 file/Novel_Structure/LP42A2/Novel_353.pdf]

Provisional ID : NC_019463.2_14426
Score total : 0.5
Score for star read(s) : -1.3
Score for read counts : 0
Score for mfe : 0.2
Score for randfold : 1.6
Score for cons. seed :
Total read count : 16
Mature read count : 16
Loop read count : 0
Star read count : 0

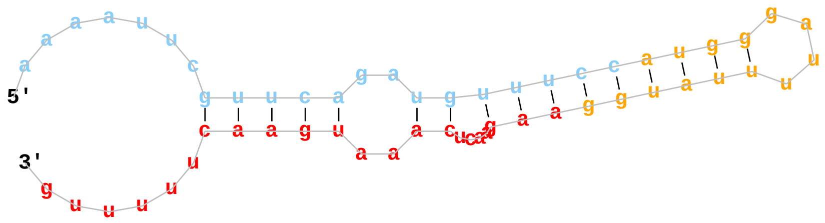

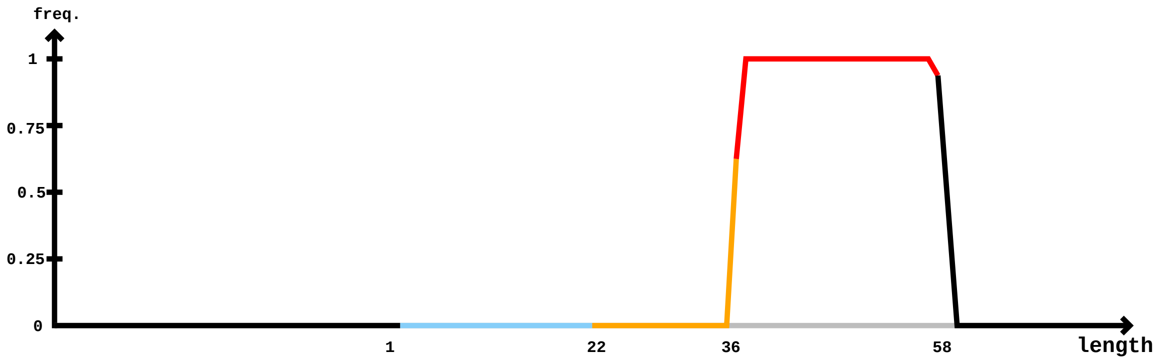

Star Mature

```
5'- gacagugguaaaaagcuauauacugggcuggccaaaaauucguucagauguuuccaugggauuuuauggaagaacucaaaugaacuuuuuggucaauucaauauuugcuu   -3'    exp
    ............((((...(((.((((.((((((((((((((((......(((((((((....)))))))))......)))))).)))))))))).)))).)))..))))    reads      mm         sample
    .....................................................................aaAaacucaaaugaacuuuuu....................    1          1          seq
    .....................................................................aaAaacucaaaugaacuuuuug...................    9          1          seq
    ......................................................................aAaacucaaaugaacuuuuug...................    6          1          seq
```
